# Unwinding forward and sliding back: an intermittent unwinding mode of the BLM helicase

**DOI:** 10.1093/nar/gkv209

**Published:** 2015-03-12

**Authors:** Shuang Wang, Wei Qin, Jing-Hua Li, Ying Lu, Ke-Yu Lu, Da-Guan Nong, Shuo-Xing Dou, Chun-Hua Xu, Xu-Guang Xi, Ming Li

**Affiliations:** 1 Beijing National Laboratory for Condensed Matter Physics and CAS Key Laboratory of Soft Matter Physics, Institute of Physics, Chinese Academy of Sciences, Beijing 100190, China; 2 College of Life Science and Technology, Northwest A & F University, Yangling, Shaanxi 712100, China; 3 Laboratoire de Biologie et PharmacologieAppliquée, Ecole Normale Supérieure de Cachan, Centre National de la Recherche Scientifique, 61 Avenue du Président Wilson, 94235 Cachan, France

## Abstract

There are lines of evidence that the Bloom syndrome helicase, BLM, catalyzes regression of stalled replication forks and disrupts displacement loops (D-loops) formed during homologous recombination (HR). Here we constructed a forked DNA with a 3′ single-stranded gap and a 5′ double-stranded handle to partly mimic a stalled DNA fork and used magnetic tweezers to study BLM-catalyzed unwinding of the forked DNA. We have directly observed that the BLM helicase may slide on the opposite strand for some distance after duplex unwinding at different forces. For DNA construct with a long hairpin, progressive unwinding of the hairpin is frequently interrupted by strand switching and backward sliding of the enzyme. Quantitative study of the uninterrupted unwinding length (time) has revealed a two-state-transition mechanism for strand-switching during the unwinding process. Mutational studies revealed that the RQC domain plays an important role in stabilizing the helicase/DNA interaction during both DNA unwinding and backward sliding of BLM. Especially, Lys1125 in the RQC domain, a highly conserved amino acid among RecQ helicases, may be involved in the backward sliding activity. We have also directly observed the *in vitro* pathway that BLM disrupts the mimic stalled replication fork. These results may shed new light on the mechanisms for BLM in DNA repair and homologous recombination.

## INTRODUCTION

The RecQ family helicases have been highly conserved during evolution from bacteria to human. Defects in three of the human RecQ members give rise to defined genetic diseases that are characterized with cancer predisposition and/or premature aging. The disorders are Bloom (BLM), Werner (WRN) and Rothmund-Thomson syndromes, caused by loss-of-function mutations in BLM, WRN and RECQ4 helicases, respectively (1–3). BLM is a DNA structure-specific helicase that unwinds DNA in 3′-5′ direction (4,5), and shows an apparent preference for substrates like Holliday junctions, G-quadruplexes, DNA displacement loops (D-loops) and stalled replication forks (6–12). These substrates represent different DNA structures that can be formed *in vivo* during DNA replication and homologous recombination (HR). It has become evident in recent years that HR and repair of stalled replication forks are intimately connected and, in many cases, loss of function of a helicase can have an adverse effect on both HR and replication fork management (13–16).

According to its amino acid sequence, BLM consists of an N-terminal region, the helicase core (BLM^642–1290^) and a C-terminal region (17). The N-terminal region mediates the oligomerization of BLM, while the function of the C-terminal region remains unclear (18). The helicase core, whose crystal structure in complex with a partial duplex DNA has been obtained recently (19), possesses a DNA unwinding activity similarly to the full length enzyme (20). BLM shares three conserved sequences with other RecQ family members, including DEAH helicase domain, RQC domain and HRDC domain (17,21). The DEAH helicase domain is composed of seven conserved motifs that form the core of two RecA-like domains which bind the 3′-ssDNA tail of a DNA fork and act as an adenosine triphosphate (ATP)-dependent DNA translocation module (22,23). The RQC domain is composed of two sub-domains: a Zn^2+^-binding domain and a so-called winged-helix (WH) domain, which plays an important role in the specific binding of the helicase for replication fork, Holliday junctions and G-quartet structures (24–29). The crystal structure reveals that the RQC domain also plays an important role in DNA unwinding by interacting directly with the duplex part of the DNA substrate (19). The binding affinity of the HRDC domain for ssDNA is markedly low (30,31), it was speculated that the spatial position of the HRDC domain may be involved in Holliday junction disruption (26).

Many investigations have contributed to the understanding of BLM-mediated DNA transactions. The BLM helicase catalyzes the regression of a replication fork, which is an important intermediate during DNA replication (11,12). It also binds D-loops specifically and plays several putative roles in the disruption pathways of D-loops (7,10,32,33). In spite of the intensive studies, the underlying molecular mechanisms, however, remain elusive. Recently, a single-molecule-fluorescence study has revealed that BLM is able to ‘measure’ how many base pairs it has unwound. Once it has unwound a critical length, it then returns and reinitiates new unwinding events in a highly repetitive fashion (34). This astonishing behavior has not yet been well interpreted, making the BLM helicase even more mysterious.

In this work, we used magnetic tweezers (MT) to study the unwinding mechanism of a fragment of human BLM, BLM^642–1290^, in more details and found some interesting behaviors of the BLM helicase. We observed a fine intermittent unwinding mode for BLM, in which the enzyme's unwinding process was disrupted by strand switching very frequently. The enzyme tends to slide rather than translocating along the opposite strand after switching strand. Quantitative analysis implies a two-state-transition mechanism for the kinetics of 3′ to 5′ strand switching. With a substrate that partly mimics a stalled replication fork, we observed the pathway in which BLM functions on a stalled replication fork *in vitro*. The involved processes are closely related to the abovementioned model. In addition, mutational studies show that Lys1125 in the RQC domain plays an important role in the backward sliding activity of BLM.

## MATERIALS AND METHODS

### Protein and DNA substrates

The BLM helicase and the mutants were prepared as described (4,35). Three types of DNA substrates (with 40-bp, 45-bp and 270-bp hairpins) were constructed with the method reported by Luzzietti *et al* (36), in which nicking enzymes were used (See Supplementary data for details).

### Single-molecule assay

DNA unwinding experiments were carried out with magnetic tweezers. A flow chamber was assembled with a slide and a cover slide, and was placed on an inverted microscope (IX71, Olympus). Both slides were cleaned and the cover slide was modified with anti- digoxigenin proteins. A magnetic bead was tethered to the modified cover slide through a single DNA substrate, before injecting a reaction buffer of 25 mM Tris-HCl (pH 7.5 at 25°C), 100 mM NaCl, 1 mM MgCl_2_, 0.1 μg/μl BSA, 1 mM ATP and 3 mM DTT to remove free substrates and beads. An external magnetic force was applied on the magnetic bead by placing a permanent magnet above the chamber. The distance between magnetic bead and the cover slide, that is, the DNA extension, was monitored by analyzing the shape of diffraction rings of the magnetic bead (37). The shape of diffraction rings depends on the distance between the bead and the focal plane of the objective (100×, NA 1.45, Olympus). A stack of calibration images that recorded the shape of the diffraction rings versus distance was obtained by stepping the focal plane through a series of positions. After checking the state of the DNA-magnetic bead connection, the BLM helicase was injected at a concentration of 5–20 nM. The DNA extension versus time was monitored.

### Data analysis

The DNA unwinding and rewinding events were monitored by recording the magnetic bead-surface distance, i.e. the DNA extension, as a function of time. The DNA extension change at a certain force can be converted into the number of base pairs unwound by using the force versus extension curve of single-stranded DNA which is well described by Freely Jointed Chain (FJC) model at low forces (38).

## RESULTS

### Elementary unwinding signals of BLM are grouped together

**Figure 1.**
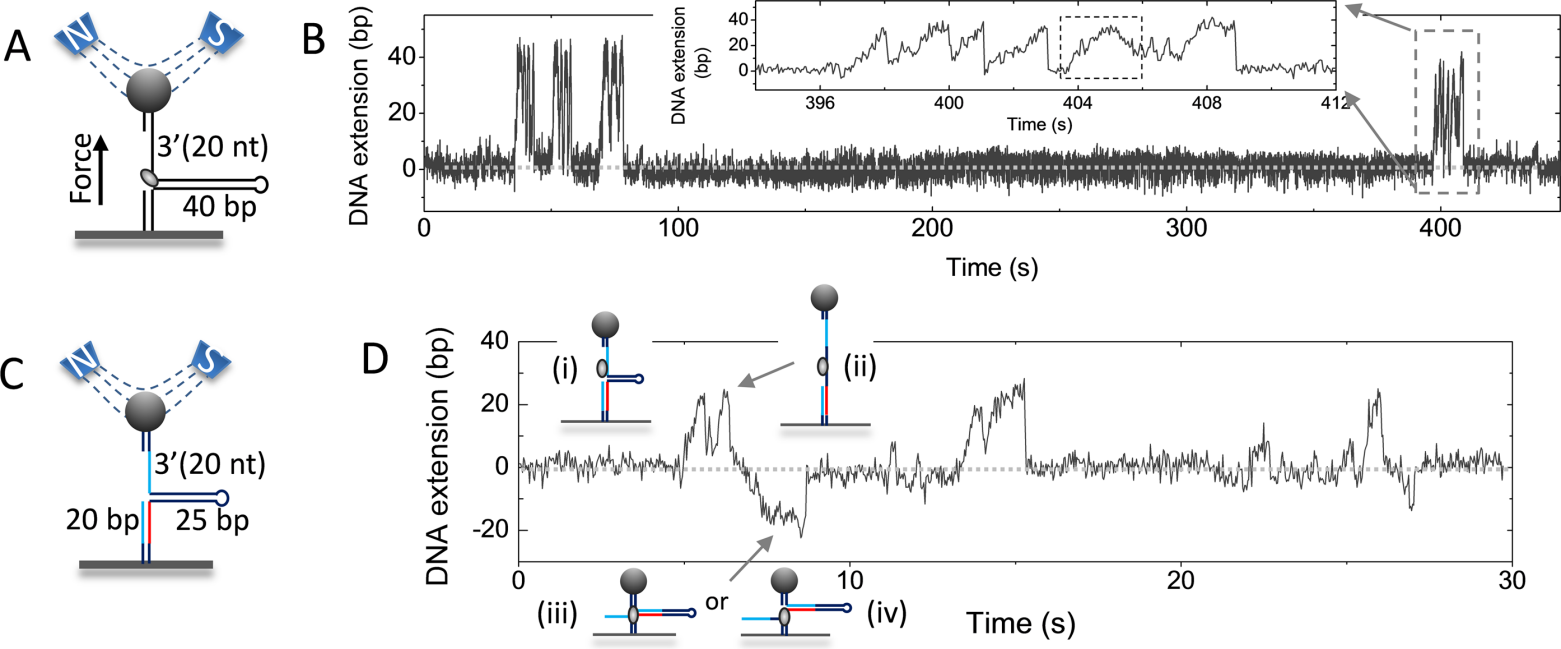
Unwinding of DNA with a short hairpin measured by using magnetic tweezers. (**A**) A magnetic bead was tethered to the glass surface of a fluid chamber through a DNA substrate with a 40-bp hairpin. The substrate was constructed with a 20-nt 3′ single-stranded gap for the binding of BLM. (**B**) DNA extension versus time in the presence of 5 nM BLM and 1 mM ATP. Each burst of oscillations consists of several elementary unwinding signals (inset). In most cases, an elementary unwinding signal is characterized by a slow increase (or unwinding process) and then abrupt decrease (or rezipping process) of DNA extension. Occasionally, an elementary unwinding signal with a slow rezipping process is observed (marked by a dashed rectangle). (**C**) The DNA construct mimicking a stalled replication fork. The red ssDNA segment is complementary to the two blue ssDNA segments. (**D**) Time trace of DNA unwinding with the mimic DNA in the presence of 5 nM BLM and 1 mM ATP. Also shown are the different states of the DNA construct that correspond to the different extensions.

We first measured the helicase activity of BLM on a forked DNA with a 40 base-pair hairpin by using MT (Figure 1A). A magnetic bead was tethered to the bottom surface of a flow chamber through two duplex handles of the DNA construct. The hairpin is stable under an external stretching force of less than 14 pN. Beyond that, the DNA extension increases abruptly, indicating that the hairpin is unzipped by force as reported previously (39). If the force is around 14 pN, the hairpin hops between the folded and unfolded states (Supplementary Figure S1). It is by this way that we check if the magnetic bead is tethered to the surface through a single DNA construct or not. When a BLM molecule loads to the 3′-ssDNA gap and unwinds the hairpin, 2 nucleotides (nt) will be released per base pair (bp) unwound.

We have observed that, when the stretching force is set between 4 and 13 pN and the concentration of BLM is lower than 5 nM, the DNA extension displays bursts of oscillations, with each burst consisting of a series of elementary unwinding signals (Figure 1B and Supplementary Figure S2). An elementary unwinding signal is characterized by a gradual increase followed by an abrupt decrease of the DNA extension. The former is attributed to the enzyme-catalyzed unwinding of the hairpin. The latter should be caused by spontaneous rezipping of the unwound DNA after the enzyme switches strands and slides back (this will be discussed later). The rezipping process is so fast that its rate cannot be determined by our instrument (with a time resolution of 30 ms). The oscillation bursts are separated by time intervals varying from several to more than one hundred seconds. When the enzyme concentration is decreased, the main feature of the unwinding behavior does not change except that the time intervals increase significantly. Therefore, the grouping of the elementary unwinding signals suggests that the short forked DNA is repeatedly unwound by a single enzyme molecule. The time interval should correspond to the time required for rebinding of another enzyme molecule. These results are consistent with previous observations using single-molecule fluorescence resonance energy transfer (smFRET) assay (34). However, the unwinding/rezipping cycle in the present work does not repeat as many times as that in the smFRET measurement (34), probably because of the different DNA constructs and assays. The DNA substrates are simply forked duplex DNA in the previous smFRET study (34), while our DNA substrates have additionally a duplex handle. As will be seen below, the helicase may unwind the duplex handle after rezipping of the hairpin.

### BLM unwinds the lagging arm after rezipping of the opened fork

To study the relevance of BLM repetitive unwinding to its biological function and the difference between the results of Yodh *et al* (34) and ours, we then constructed a forked DNA which mimics a stalled replication fork. The new DNA construct has a 20-nt 5′ flap that is complementary to the first 20-nt segment (red) of the bottom strand of the hairpin (Supplementary Figure S3). After the DNA was tethered to the glass surface, a stretching force larger than 16 pN was applied to unzip the hairpin completely. The 5′ flap annealed spontaneously with the abovementioned 20-nt segment. The force was then reduced to 10 pN to let the remaining ssDNA form hairpin again. As a result, the DNA construct rested in a conformational state (Figure 1C, designated as State (i) in Figure 1D) of a 25-bp hairpin with a 20-bp duplex on the lagging arm (lower handle) and a 20-nt 3′ ssDNA gap on the leading arm (upper handle).

As before, upon addition of 5 nM BLM and saturating ATP (1 mM), we observed unwinding signals (Figure 1D). When the hairpin was completely disrupted (State (ii) in Figure 1D), the DNA extension reached a peak value. We noticed, however, that when the DNA extension dropped to the baseline and the DNA conformation returned to State (i), the DNA extension in some cases (∼30% of the observed events) did not stop there but continued to decrease below the baseline. It indicates that the 20-bp duplex on the lagging arm was unwound by the enzyme immediately after the disrupted hairpin rezipped. In these cases, the DNA extension decreased at a rate similar to the rate of unwinding above the baseline. It suggests that the 20-nt 5′ flap on the lagging-arm was gradually displaced by the enzyme while the exposed opposite ssDNA (red) annealed spontaneously with its complementary part in the 20-nt 3′ ssDNA gap on the upper duplex handle, resulting in a DNA conformation in State (iii). Accordingly, the DNA extension decreased at a rate determined by the duplex unwinding rate of the enzyme. These results demonstrate that the enzyme has the capacity of unwinding the lower dsDNA handle after the hairpin is unwound in the previous experiment with the 40-bp-hairpin substrate (Figure 1A), and this is possibly the reason for the difference between the results of Yodh *et al* (34) and ours.

The enzyme may continue to unwind the lower duplex handle after the 20-nt 5′ flap is displaced completely, but this will induce no obvious change in DNA extension because no more DNA annealing occurs (State (iv)). The extension therefore shows a pause at about 20 nm below the baseline even though the enzyme may continue to unwind the DNA. Finally, the pause is ended by the DNA extension quickly jumping back to the baseline, indicating that the DNA structure spontaneously returns from States (iii) or (iv) to State (i). We think this phenomenon can be explained as follows. Under a 10-pN stretching force, though the hairpin structure in States (iii) or (iv) cannot be disrupted, the duplex base-pairing of the hairpin near the junction may be disrupted temporarily by the stretching force. The consequence of this structural fluctuation is that, once the enzyme dissociates or switches strands, the complementary 5′ flap may anneal with the unzipped ssDNA (red) to form part of the lower duplex handle again. The new formed duplex is more stable because the stretching force does not affect its structure. Thus the DNA returns to its starting structure (State (i)) with its extension jumping back to the baseline. If the jump is caused by enzyme dissociation, DNA unwinding will reinitiate only after the binding of another enzyme. If the jump is caused by strand switching of the enzyme, depending on the position of the enzyme, unwinding of the hairpin or the lower handle may reinitiate immediately. The former leads to increase of DNA extension above the baseline while the latter to decrease of DNA extension below the baseline (Supplementary Figure S4A). These unwinding processes may repeat for several times. In some cases (less than 6% of the observed events), the DNA extension decreases directly below the baseline from the very beginning of an unwinding burst (Supplementary Figure S4B), suggesting that the enzyme may initially bind and unwind the lower handle directly.

### Unwinding signal of long hairpin consists of unwinding slopes interrupted by rezipping jumps

**Figure 2.**
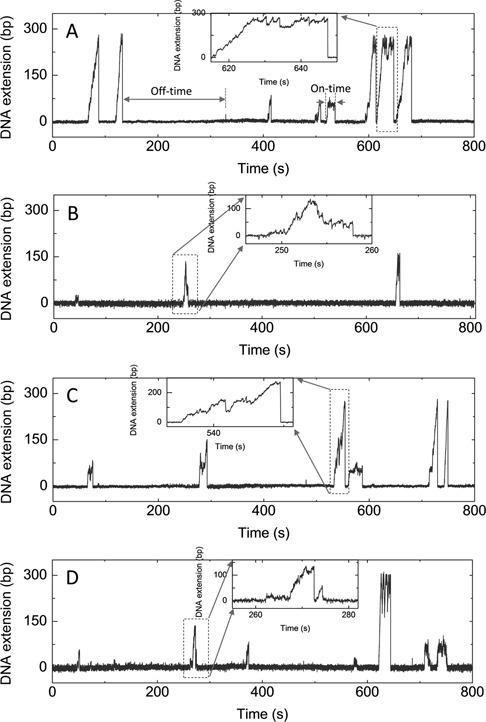
Unwinding of the 270-bp hairpin by the wild-type and mutant BLM (5 nM) at 1 mM ATP. (**A**) DNA extension versus time for the wild-type BLM. Two time parameters are defined. The on-time is defined as the duration of a burst of unwinding events, i.e. from unwinding initiation until the DNA extension falls back to the baseline without further immediate unwinding. The off-time is defined as the time interval between two adjacent bursts of unwinding. (**B**−**D**) DNA extension versus time curves for T1110G (B), S1121A (C) and K1125A (D), respectively.

To confirm that the short unwinding lengths in the previous experiments (Figure 1 and Supplementary Figure S2) are not due to the short hairpin, we constructed a forked DNA with a long hairpin (270 bp). Again, the unwinding signals are grouped together. Some sudden drops of DNA extension appear during the progressive DNA unwinding processes (Figure 2A and Supplementary Figure S5) even under the enzyme concentration of 1 nM (Supplementary Figure S13). But here, the BLM helicase remains functioning (i.e. undissociated) on the 270 bp DNA for a longer time than it does on the 40 bp hairpin. Notably, although occurring not very frequently (less than 20% of the observed events), slow rezipping events were also observed (Supplementary Figure S6), suggesting that the enzyme is translocating on the opposite strand at a rate equal to its unwinding rate rather than slipping in these cases.

To quantitatively analyze the unwinding processes, we introduce two parameters, on-time and off-time (Figure 2A). The on-time is defined as the duration of a burst of unwinding events, i.e. from unwinding initiation until the DNA extension falls back to the baseline without further immediate unwinding. It should correspond to the time during which the BLM helicase remains attached to the DNA substrate. The off-time is defined as the time interval between two adjacent bursts of unwinding, corresponding to the time required for BLM to rebind the DNA substrate. The distributions of on- and off-times at two different BLM concentrations are shown in Supplementary Figure S7. All distributions decay exponentially. The decay time constant (*t*_on_) of the on-time distribution is almost independent of the BLM concentration, while that (*t*_off_) of the off-time distribution decreases significantly from 62.9 to 20.7 s as the BLM concentration increases from 5 to 10 nM. The exponential decay of the on-time distribution indicates that the dissociation of the enzyme is characterized by a Poisson process. It implies that only one molecule is working on the substrate, which agrees with previous results (5,40). We found that if the BLM concentration is much higher than 10 nM, the on-time distribution deviates from the single-exponential decay. This is because more than one molecule may bind to the substrate simultaneously.

### BLM unwinds DNA substrates uninterruptedly, which is reinitiated immediately after fast rezipping

**Figure 3.**
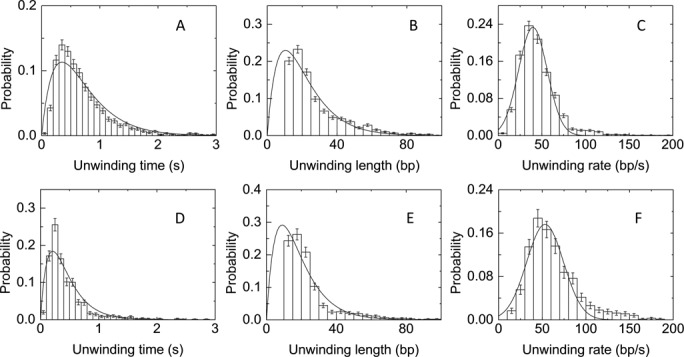
Distributions of unwinding parameters for BLM-WT (**A**−**C**) and K1125A (**D**−**F**) at 5 nM enzyme, 1 mM ATP and 12 pN stretching force. (A, D) Distributions of unwinding time. They are fitted with Supplementary Equation (1), yielding *k*_1_ = 3.16 ± 0.09 s^−1^, *k*_-1_ = 0 s^−1^, *k*_2_ = 3.28 ± 0.10 s^−1^ (*n* = 2176 events) (A), and *k*_1_ = 3.91 ± 0.61 s^−1^, *k*_-1_ = 0 s^−1^, *k*_2_ = 10.47 ± 1.91 s^−1^ (*n* = 879 events) (D), respectively. (B, E) Distributions of unwinding length. They are fitted with Supplementary Equation (2), yielding *k*_1_/*R*_unwind_ = 0.062 ± 0.005 bp^−1^, *k*_-1_/*R*unwind = 0 bp^−1^, *k*_2_/*R*_unwind_ = 0.213 ± 0.069 bp^−1^ (*n* = 2176 events) (B), and *k*_1_/*R*_unwind_ = 0.085 ± 0.016 bp^−1^, *k*_-1_/*R*_unwind_ = 0 bp^−1^, *k*_2_/*R*_unwind_ = 0.225 ± 0.060 bp^−1^ (*n* = 879 events) (E). (C, F) Distributions of unwinding rate. They are fitted with the Gaussian equation, yielding unwinding rates of 39.6 ± 1.5 bp/s (*n* = 2176 events) (C) and 53.2 ± 2.7 bp/s (*n* = 879 events) (F), respectively.

The distribution of the on-time indicates that an enzyme works for ∼23 s on average before it dissociates from the DNA substrate. However, the enzyme unwinds uninterruptedly only a very short segment of the DNA hairpin at a time (Figure 2A). To analyze the unwinding dynamics of the enzyme, we define an unwinding time which is the duration of continuous unwinding between two adjacent sudden drops of DNA extension. Due to the limited resolution of our home-made magnetic tweezers, we used a threshold in the data analyses, that is, we only collected the unwinding/rezipping signals with distance changes larger than the spatial resolution (10 bp). Unexpectedly, the distributions of the unwinding time displayed an exponential rise followed by an exponential decay (Figure 3A and Supplementary Figure S8A). According to the single-enzyme dynamic theory (41), a two-rate-transition should be involved in the unwinding processes.

Similarly, another parameter, unwinding length (*l*_unwind_), was defined as the length of DNA unwound during the unwinding time just defined above. The distributions of the unwinding length showed an obvious peak at ∼15 bp (Figure 3B and Supplementary Figure S8B). The distributions of unwinding rate (*R*_unwind_) of the BLM helicase were also obtained at the two external forces of 9 and 12 pN (Figure 3C and Supplementary Figure S8C).

### BLM displays unwinding/rezipping cycles without external force

To see if the repetitive unwinding/rezipping still occurs without external force applied to the DNA substrate, we measured the unwinding behaviors of the BLM helicase using the FRET technique. A forked DNA was constructed with a double-stranded part of 40 bp and two single-stranded tails of 15 and 26 nt, respectively. This forked DNA was tethered to the glass surface through biotin-streptavidin interaction (see Supplementary text and Figure S9A). After the injection of 5 nM BLM and 1 mM ATP, similar repetitive unwinding patterns were observed (Supplementary Figure S9B). We also studied the unwinding patterns with 5 nM BLM and 5 μM ATP, as used by Yodh *et al* (34). The patterns under these two conditions were nearly the same except for the unwinding rate. Quantitative analysis of the unwinding/rezipping cycles showed a peak around ΔFRET = ∼0.4 (Supplementary Figure S9B and C). These results confirmed the existence of a peaked distribution for the unwinding length in the experiments with magnetic tweezers. They also agree with the results of Yodh *et al* (34).

### Mutation in the RQC domain reduces the binding time of BLM on DNA

To study the roles played by the RQC domain in the repetitive unwinding activity of BLM, we chose to mutate three amino acids (Thr1110, Ser1121 and Lys1125) that are involved in the interactions between RQC and the 5′ strand in the duplex part of the DNA substrate (19). We speculate that the interaction between RQC and the 5′ strand should be essential for the strand switching and backward sliding of BLM (this will be discussed later).

**Figure 4.**
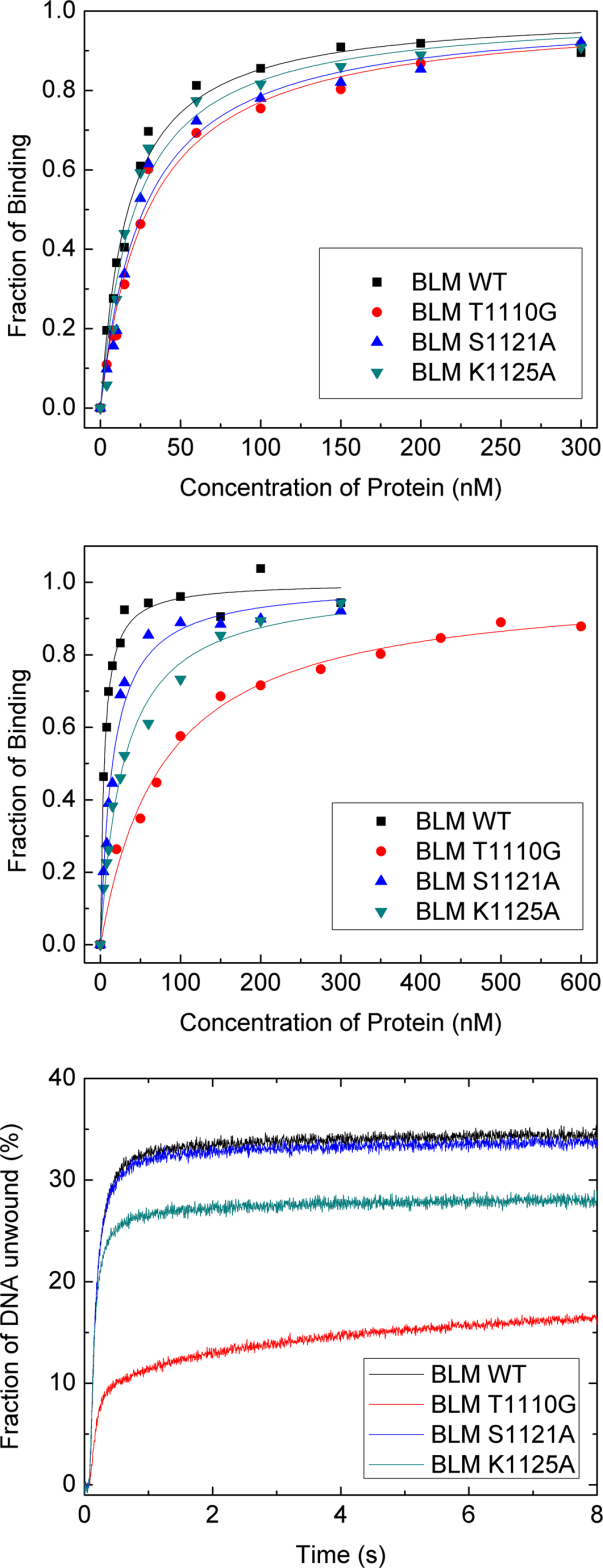
Characterizations of the DNA binding and unwinding activities of the wild-type and mutant BLM. The polarization anisotropy assay for DNA binding and stopped-flow assay for DNA unwinding were performed as described in the Supplementary data. (**A** and **B**) Binding curves for ssDNA (18 nt) (A) and dsDNA (18 bp) (B). The dissociation constants were obtained from fits with the Hill equation and given in Table 1. (**C**) Kinetic DNA unwinding curves.

DNA binding measurements showed that these mutants have similar binding affinities for ssDNA as the wild-type enzyme (Figure 4A and Table 1), whereas their binding affinities for dsDNA are more significantly reduced (Figure 4B and Table 1). From the dissociation constants, the binding affinities for dsDNA are in the following order: wilde-type > S1121A > K1125A > T1110G. DNA unwinding efficiencies from further stopped-flow kinetic measurements essentially agree with the binding results, indicating that the RQC domain plays an important role in the DNA unwinding activity of BLM.

**Table 1.** Dissociation constants for the wild-type and mutant BLM in binding ssDNA and dsDNA

Protein	*K*_d_ (nM)	
	ssDNA	dsDNA
BLM^642–1290^	17.2 ± 1.6	4.6 ± 0.5
BLM^642–1290^ T1110G	29.7 ± 3.6	78.2 ± 7.2
BLM^642–1290^ S1121A	27.1 ± 3.7	14.9 ± 1.7
BLM^642–1290^ K1125A	21.3 ± 2.9	28.6 ± 2.6

To see how these mutations affect the unwinding behaviors of BLM at the single-molecule level, we used magnetic tweezers to study their unwinding processes with the same 270-bp hairpin DNA substrate as before. Typical time traces of DNA length in the presence of the 5 nM BLM mutants and 1 mM ATP are shown in Figure 2B−D.

Among the three mutants, T1110G only unwinds the DNA substrate occasionally, that is, we observed only three unwinding bursts in 800 s (Figure 2B). Similar unwinding behaviors were observed when increasing the enzyme concentration to 20 nM. S1121A unwinds the DNA substrate with the same unwinding/rezipping behavior as the wild-type enzyme does (Figure 2C). K1125A also unwinds DNA substrate in the same repetitive way as the wild type does, but less repetitively: about 30% of the unwinding bursts have only one unwinding/rezipping cycle which rarely occurs in the case of the wild-type enzyme under the same conditions.

The distributions of on-time (duration of an unwinding burst) for S1121A and K1125A are given in the Supplementary Figure S10. Both histograms can be well fit with a single-exponential decay, as in the case for the wild-type enzyme. The time constants (average on-times) for the two mutants are 16.8 and 6.2 s, respectively, which are lower than that for the wild-type enzyme, 25.5 s (Supplementary Figure S7). We cannot obtain the on-time distribution for T1110G due to the rare occurrence of unwinding events, but it is clear from Figure 2 that the average on-time for T1110G should be even lower than the other two mutants. These on-time results for the wide-type and mutant enzymes correlate with their affinities for dsDNA, indicating that the interaction between the RQC domain and the DNA duplex is indispensable for the stabilization of the complex BLM/DNA substrate during duplex unwinding.

### Mutation in the RQC domain does not affect the unwinding dynamics in each unwinding/rezipping cycle

Distributions of the unwinding time, unwinding length and unwinding rate in the unwinding/rezipping cycles were obtained at the enzyme concentration of 5 nM and under the external force of 12 pN for K1125A (Figure 3D−F) and S1121A (Supplementary Figure S11A−C). Histograms of unwinding time showed similar peaks at ∼0.3 s. Histograms of unwinding length showed similar peaks at about 15 bp. Also histograms of unwinding rate showed similar peaks at about 50 bp/s. All values are similar to the corresponding ones for the wild-type enzyme (Figure 3A−C). These results indicate that the enzyme tends to unwind the DNA substrate for relatively fixed time interval and length before switching strand, regardless of the strength of interaction between the RQC domain and the 5′ ssDNA strand in the duplex part of the DNA substrate. We cannot do the same analysis for the T1110G mutant because very few data were collected due to its low DNA unwinding activity. But the unwinding/rezipping behaviors in the few unwinding bursts we observed seem to still support the above conclusion. It should be noted that this conclusion does not mean that the RQC domain is not involved in the sliding process of the enzyme after it switches strand.

### K1125A dissociates more easily during the sliding process

As has been mentioned before, K1125A sometimes (about 30% of the unwinding bursts) unwinds the DNA substrate for only one unwinding/rezipping cycle in an unwinding burst. This phenomenon rarely occurs with the wild-type enzyme or S1121A. Even with the T1110G, repetitive unwinding behavior still occurs in the few bursts that were observed. We analyzed the unwinding lengths in such unwinding bursts (with only one unwinding/rezipping cycle), single-cycle unwinding lengths (Supplementary Figure S12A), and found that K1125A preferred an unwinding length similar to the unwinding lengths in the normal unwinding bursts (Figure 3E). This implies that that K1125A falls off the DNA substrate after switching strand. Thus, though the mutation at Lys1125 does not affect the strand-switching activity, it affects the following backward sliding of BLM. That is, Lys1125 is critical for the backward sliding activity of BLM.

Another proof for this observation is that K1125A unwinds the lower handle with a probability of only ∼6% after hairpin rezips, whereas, as mentioned before, the probability is ∼30% for the wild-type enzyme (Figure 1C and D). This decrease should result from the loss of interaction between Lys1125 and the DNA substrate, which is involved in the backward sliding of the enzyme.

## DISCUSSION

### BLM slides back after strand switching

The distributions in Figure 3A and B indicate that the BLM helicase tends to unwind DNA for a preferred number of base pairs, ∼15 bp, which agrees with the processivity of BLM as determined by stopped-flow assay (5). In the previous smFRET assay, however, the number of base pairs unwound in each unwinding cycle was not determined exactly because the FRET signal change could not be scaled quantitatively with the unwinding length, and was only estimated to be less than 34 bp (34). The low processivity of BLM may arise from its high ssDNA release rate in the adenosine diphosphate (ADP) state (5,42). Our present study indicates that the application of an external force does not affect the processivity of BLM. Furthermore, it is shown that BLM does not dissociate from the substrate readily. Instead, several elementary unwinding events often happen consecutively, suggesting a single BLM helicase may work on the substrate for a long time and unwind accumulatively a long run of DNA duplex.

Note that the elementary unwinding signal of BLM exhibits a feature of gradual unwinding of duplex DNA interrupted by abrupt rezipping, which is different from the results reported by Yodh *et al* (34). The abrupt rezipping phenomenon observed here cannot be attributed to the translocation of the enzyme on the opposite strand because this would result in a slow rewinding of the hairpin with a rate similar to that of unwinding (42), as was also observed in our experiments (Supplementary Figure S6). The abrupt rezipping can be explained, however, by the slippage of the enzyme on ssDNA, either the tracking strand or the opposite strand (see below). This may occur if the enzyme binds loosely to the ssDNA so that it can be pushed to slide back by the spontaneously rezipping hairpin. For example, the AtRECQ2 helicase can have a slippage in the direction of its unwinding polarity (43), while the T7 helicase can slide in both directions (44).

The following two results indicate that the BLM helicase slides back on the opposite strand (after switching strand) rather than on the tracking strand. (i) Slow rewinding events that arise from translocation of the enzyme on the opposite strand were occasionally observed and a slow rewinding event is often followed immediately by a fast rezipping event, or vice versa (Supplementary Figure S6). (ii) In the experiments with a 5′ flap (Figure 1D), the enzyme unwinds the lower handle immediately after the hairpin rezips. This can be easily explained if the enzyme is sliding back on the opposite strand during rezipping of the hairpin. Otherwise, if the enzyme slides back in the 5′ to 3′ direction on the tracking strand, it will reinitiate unwinding of the hairpin again.

A possible explanation of the slippage may involve the rezipping of the unwound hairpin. It is reasonable to assume that the enzyme always binds to ssDNA, either the tracking strand or the opposite strand, in the 3′-5′ direction. When the enzyme is unwinding the hairpin, the fork exerts a resistance on the enzyme. When the enzyme is translocating on the opposite strand, the fork pushes the enzyme from behind. In both cases, BLM should slip along ssDNA as soon as it is in a weak DNA binding state during ATP hydrolysis (5,42). But why BLM only slides back on the opposite strand? We think the reason is that, during unwinding, BLM binds not only to the tracking ssDNA, but also to the duplex at the ss/dsDNA junction (19,24,26). The latter binding site may inhibit the backward slipping of the enzyme during unwinding.

In the previous smFRET study (34), the reannealing was gradual and depended on ATP concentration and thus was attributed to the ATP-driven translocation of the enzyme on the opposite strand. But we have revealed that BLM slides back rather than translocating on the opposite strand during the reannealing of hairpin. It is obvious that the enzyme unwinds and slides on the dsDNA handle in a similar manner (Supplementary Figure S4) though the DNA substrate is similar to the forked one in the smFRET experiments (34). Note that smFRET technique is known for its high spatial resolution in the range of 3–8 nm but it is not practical for direct distance detection compared with magnetic and optic tweezers. For instance, when the FK34 substrate was unzipped and the distance between donor and acceptor exceeded the sensitive detection range, the FRET signal would not make clear distinction between translocation or sliding (34).

### A single BLM can function on a stalled replication fork

**Figure 5.**
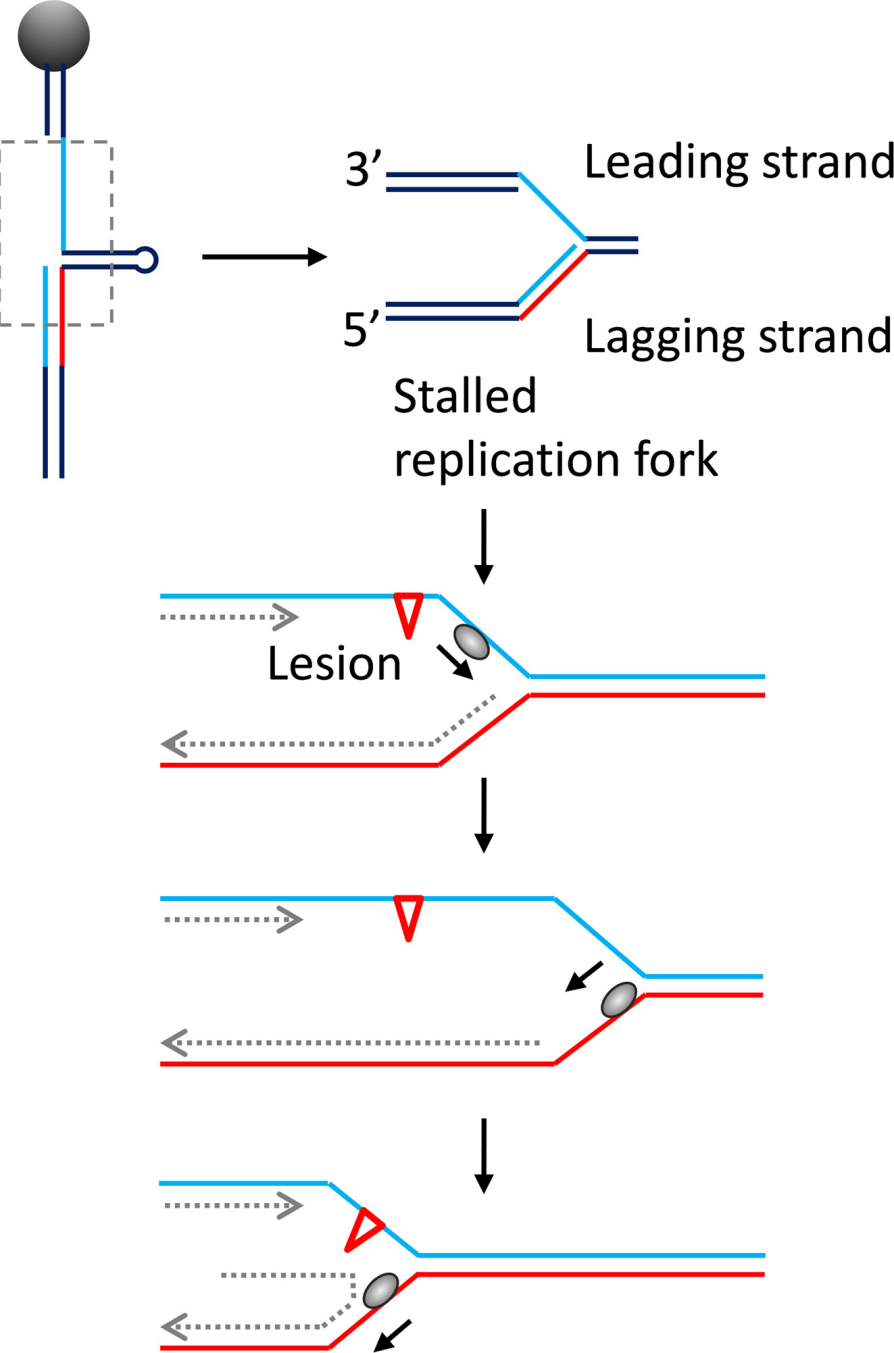
The functions of BLM in repair of stalled replication forks. The 25-bp hairpin construct mimics a stalled replication fork. BLM unwinds the stalled replication fork and subsequently the duplex on the lagging arm in a repetitive manner. As a result, a single lagging strand is released.

BLM plays important roles in both HR and repair of stalled replication forks. The construct resembles a stalled replication fork (Figure 5) which can be caused by many types of DNA damages (11,45). Our present single-molecule study showed that a single BLM helicase can generate a single stranded tail on a stalled replication fork and provided a three-step mechanism for this flap switching function by directly observing its unwinding process on stalled replication fork (Figure 5): (i) a BLM molecule binds the single-stranded gap on the leading template and unwinds the parental DNA duplex ahead of the fork to eliminate the structures that impede the replication machinery; (ii) after unwinding a small number of base pairs, the enzyme switches to the opposite strand and slides back quickly. The above two processes may repeat several times until the enzyme catches the ss-dsDNA junction on the lagging arm; (iii) the enzyme unwinds the duplex on the lagging arm and releases the lagging strand. It should be mentioned that, basing on biochemical and genetic studies, a simpler two-step model has been proposed previously by Hishida *et al* (46) for the probable role of *E. coli* RecQ in SOS signaling and genome stabilization. Due to the highly conserved amino sequences and the similar functions involved in genome stabilization of the RecQ family helicases, this three-step mechanism for BLM may give some implication to *E. coli* RecQ or other helicases of the same family and facilitate better understanding of their cellular functions.

### Molecular mechanism for the intermittent unwinding of BLM

The 40-bp hairpin (Figure 1A) is not a good substrate for the study of the strand-switching mechanism because the hairpin is not much longer than the preferred unwinding length. We therefore studied the unwinding of a 270-bp hairpin at various forces (Figure 2). In this case, the progressive unwinding of the DNA duplex by the enzyme is frequently interrupted by abrupt rezipping of the unwound DNA. Sometimes, a long run of DNA duplex and even the whole hairpin can be unwound without any interruption by rezipping. This is different from the observation in the previous smFRET study (34), where the BLM helicase unwinds DNA substrates no more than 34 bp in each unwinding/reannealing cycle.

**Scheme 1.**
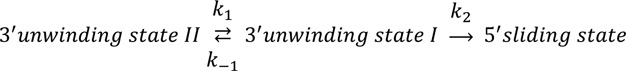


Distributions of the uninterrupted unwinding length and unwinding time of the wild type enzyme were analyzed. Histograms of the unwinding length showed an obvious peak, and the distribution of unwinding time can be described by Supplementary Equation (1) which is characteristic of an exponential rise followed by an exponential decay (Figure 3A). According to the theory of single-molecule enzyme kinetics (47,48), such a distribution implies a two-rate-limiting transition scheme (Scheme 1) for the strand-switching mechanism of BLM,

**Figure 6.**
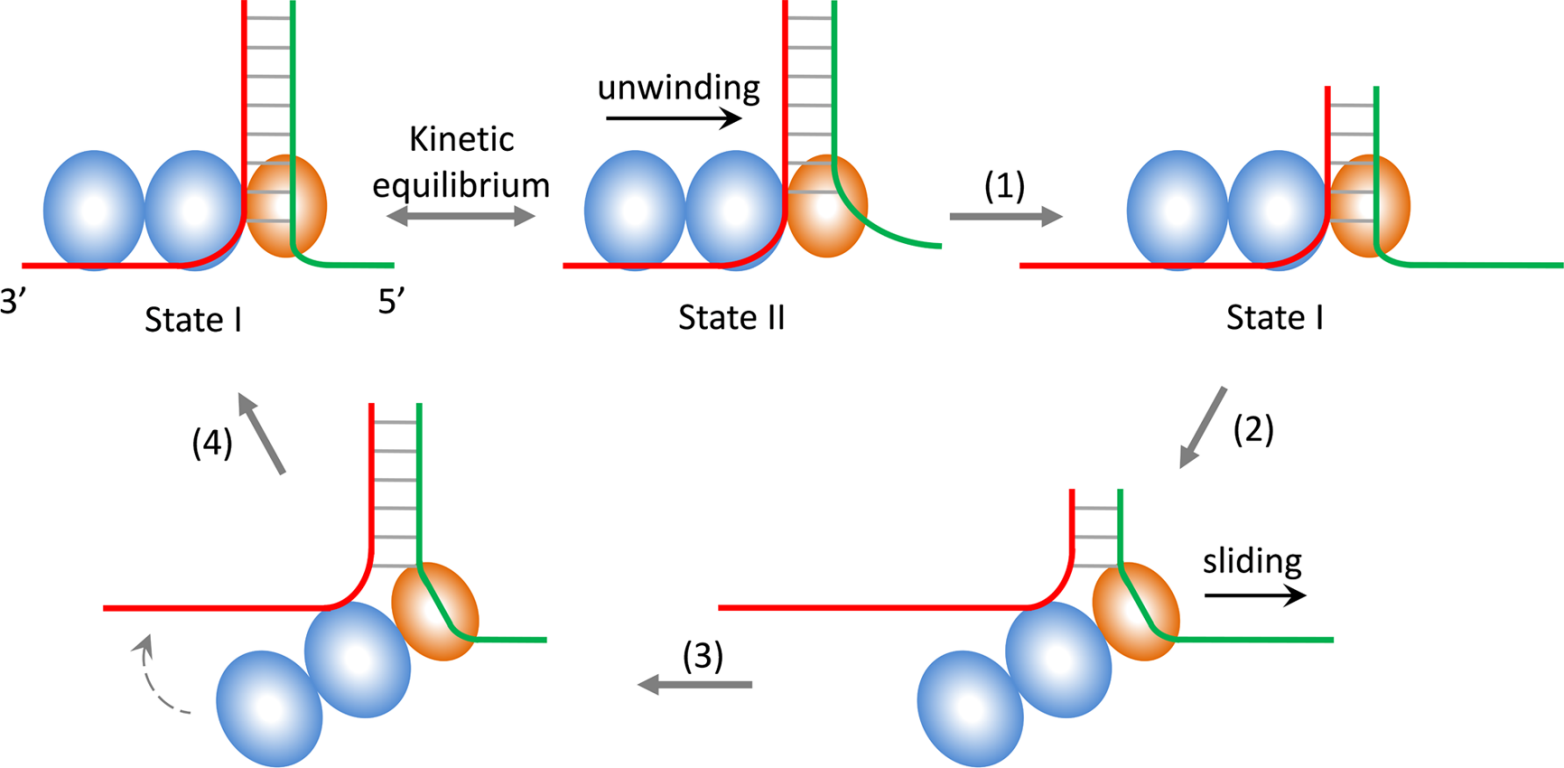
Intermittent unwinding model for BLM. The two RecA-like domains (blue) bind to the 3′ single-stranded tail (tracking strand). In the unwinding state, the RQC domain (orange) binds to both the double-stranded DNA and the 5′ extension (opposite strand). After unwinding for a certain length (step 1), the enzyme switches strand (step 2) from the tracking strand to the opposite strand upon release of the two RecA-like domains from the 3′ ssDNA. Once the enzyme switches strand, it slides back quickly along the opposite strand as is pushed by the rezipping duplex (step 3). During its backward sliding, the enzyme may bind the tracking strand (step 4) again and then restart the unwinding of the DNA duplex. These unwinding/sliding processes may repeat until the duplex is unwound completely or the enzyme dissociates from the DNA substrate.

Very recently, Swan *et al*. obtained the crystal structure of BLM in complex with ADP and duplex DNA (19). The two RecA-like domains bind the 3′ single-stranded tail and translocate along it while unwinding the duplex. The RQC domain is the primary dsDNA-binding site in BLM. It is known that the RQC domain also binds the 5′ strand of the forked DNA to be unwound (23,49).

Based on the crystal structure of BLM in complex with ADP and duplex DNA, we chose three mutation sites (S1121A, K1125A and T1110G) in the RQC domain that are involved in the interactions between the enzyme and the 5′ strand of the duplex DNA. It was found that the reduction of dsDNA binding affinity does not influence obviously the strand switching activity of BLM, because these mutants still prefer to unwind a similar duplex length and then switch strands (Figure 3E and Supplementary S11B), whereas the time of binding on the DNA substrate was indeed affected by the mutations (Supplementary S10).

It is known that Lys1125 in the RQC domain of BLM is a highly conserved amino acid among RecQ helicases. After mutating this amino acid to Ala, the enzyme more readily falls off the DNA substrate after switching strand. It means that Lys1125 may be critical for the backward sliding activity of BLM.

BLM uses the two RecA-like domains to translocate along the 3′-tracking strand while using the RQC domain to catalyze the separation of the base pairs (19,23–25). As shown in Scheme 1, there should be a two-rate-limiting transition (or two unwinding states) involved in the enzyme's unwinding process before strand switching. For the existence of the two unwinding states during DNA unwinding, one possible origin is the binding or unbinding of the RQC domain to the 5′ strand extension. The mutational studies, however, excluded this possibility because the mutation does not affect the unwinding time and length. Another possible origin is the binding or unbinding of the HRDC domain to the RecA-like domains, as can be conjectured from the crystal structure (19). As the HRDC domain plays a key role in the tightly coupling between the ATPase and helicase activities of BLM (19), it is reasonable to think that the HRDC domain may modulate the interaction between the two RecA-like domains and the DNA substrate.

Our proposed model is depicted in Figure 6. The enzyme jumps randomly between the two unwinding states during the unwinding process. It is only in State I can BLM switch strand. Preliminary data fittings show that the rate constant k_1_ is much larger than k_−1_ and the latter is very close to zero, thus we actually assume k_−1_ to be zero in all data fittings. This result implies that the enzyme tends to stay in State I rather than State II. That is possibly the reason that the BLM helicase switches strand very frequently. The change between two states (I and II) does not affect the unwinding process much, which at least cannot be distinguished in the unwinding patterns. After the translocation module (i.e. the two RecA-like domains) releases the 3′-tracking strand, the enzyme switches strand by remaining associated with the DNA substrate through interactions between the RQC domain and the 5′ extension. In this configuration, the enzyme may slide on the opposite strand because it binds ssDNA loosely while the restoring DNA fork pushes the enzyme from behind. During the sliding process, the enzyme may bind the 3′ ssDNA again through the translocation module and resumes duplex unwinding. This process may repeat several times until the enzyme dissociates from the substrate completely.

Similar intermittent unwinding mode has been observed in the experiment with another RecQ family member, AtRECQ2, implying this unwinding mode may not be specific for BLM (43). Note that our present strand-switching model for intermittent unwinding by BLM is different from that given by Yodh *et al* (34). In their model, the RQC domain is assumed to remain always attached to the duplex part of the DNA substrate. From the spatial arrangements of the two RecA-like and the RQC domains in the presently available crystal structure of BLM in complex with DNA (19), it is clear that their assumption is unlikely to be true.

We propose that BLM may have evolved into the intermittent unwinding mode so that it can perform optimally in both HR and stalled replication fork repair processes. (i) The unwinding forward and backward-sliding is an efficient way for the BLM helicase to catalyze the regression of stalled forks. BLM is a 3′-5′ translocase and needs a single-stranded gap to load onto the substrate. On a stalled fork, the loading site will lead the enzyme to move in the wrong direction, i.e. away from the subject to be displaced (Figure 5). Getting back promptly ensures that the subject be displaced quickly. It can also be considered that the purpose of BLM to frequently switch strand should be the unwinding of the lagging arm of the forked DNA, which is very important in HR and DNA repair. (ii) BLM may use its strand-switching ability to displace proteins such as RPA and Rad51 during replication fork repair and HR. Such a function has been partly demonstrated by Yodh *et al* (34). The BLM helicase shuttles back and forth repetitively on the single-stranded DNA to remove the SSB proteins efficiently. Also, the strand-switching activity might be a good way to keep the enzyme working on the subject for a long time, which may ensure the regression of stalled replication fork being well completed. (iii) During the early stages of HR, BLM works in conjunction with mismatch repair (MMR) machinery (50) to check for homology. BLM should not be highly processive because the invading strand is not long at an early point of HR. A highly processive helicase would disrupt most of the alignments rashly even without the stimulation of MMR machinery.

## SUPPLEMENTARY DATA

Supplementary Data are available at NAR Online.

SUPPLEMENTARY DATA
