# Supplementary material for: Unwinding forward and sliding back: an intermittent unwinding mode of the BLM helicase
Source: Nucleic Acids Res. 2015 Mar 12;43(7):3736–46. doi: 10.1093/nar/gkv209 (PMC4402530; doi:10.1093/nar/gkv209)
Supplement: SUPPLEMENTARY DATA [file supp_43_7_3736__index.html]

Unwinding forward and sliding back: an intermittent unwinding mode of the BLM helicase — Unwinding forward and sliding back: an intermittent unwinding mode of the BLM helicase — SUPPLEMENTARY DATA 

# Unwinding forward and sliding back: an intermittent unwinding mode of the BLM helicase

## SUPPLEMENTARY DATA

**Files in this Data Supplement:**

- SUPPLEMENTARY DATA
