# Supplementary material for: Unwinding forward and sliding back: an intermittent unwinding mode of the BLM helicase
Source: Nucleic Acids Res. 2015 Mar 12;43(7):3736–46. doi: 10.1093/nar/gkv209 (PMC4402530; doi:10.1093/nar/gkv209)
Supplement: SUPPLEMENTARY DATA [file supp_gkv209_nar-02355-h-2014-File008.doc]

**Supplementary Data**

**DNA substrate constructions**

All the DNA sequences listed in Tables S1−6 were purchased from Shanghai Sangon Biological Engineering Technology & Services Co., Ltd. Double-stranded DNAs (H1 and H2 sequences) were produced using the gene synthesis method. There are four BbvCI sites on H1 DNA sequence, one is at the 5' end and three are in the middle and spaced by 15 bp. H2 sequence carries one Nb.BsrDI site at the 3' end. These two DNA sequences were nicked by Nb.BsrDI and Nb.BbvCI successively, producing a single-stranded gap on H1 sequence and a single-stranded tail on each of the two sequences, and they can be hybridized and ligated to form a raw hairpin construct. Seal1 and Seal2 sequences were annealed and ligated to form a hairpin loop construct with a 10-nt tail. Annealing the hairpin loop to the raw hairpin construct and ligating them, the 270-bp hairpin DNA for helicase unwinding experiments was produced. As a result, the 270-bp hairpin DNA composed of a digoxigenin-labelled DNA end (204 bp, long enough for the separation between the duplex and the slide surface), a DNA loop region (273 bp) to be opened by BLM, and a biotinylated DNA end (115 bp) which was introduced in the PCR process of H1 DNA amplification. The 18-nt gap near the fork was designed as tracking strand for BLM. The biotinylated DNA end was tethered to neutravidin coated magnetic bead (Invitrogen, Dynabeads M-280 Streptavidin), and the digoxin modified end was tethered to an anti-digoxigenin modified surface.

The 40-bp and 45-bp hairpin was produced with similar method mentioned above by using DNA sequences in Table S2.HS1 and HS2 sequences were annealed and ligated to form a 40-nt loop, followed by hybridizing with the nicked H2 sequence and the HS4 sequence. The 45-bp hairpin was constructed by annealing with the nicked H2 sequence, the HS3 and HS4 sequences.

The forked DNA used for the single-molecule FRET assay was prepared by annealing the two sequences listed in Table S3.

**Single-molecule FRET measurements**

A wide-field total internal reflection fluorescence microscope based on an inverted microscope (IX-71, Olympus) was used to image an area of 50 μm × 100 μm to an EMCCD camera (I-PentaMAX-512EFT, Roper Scientific). Molecules were excited using a Nd:YVO_4_ laser at 532 nm (Verdi-2, Coherent) through a quartz prism placed over a quartz slide via a thin layer of immersion oil. The incident angle of the laser was controlled to achieve the total internal reflection at the interface between the quartz slide and aqueous imaging buffer. Fluorescently labeled DNA molecules are attached to this interface. Fluorescence signal was collected using a water immersion objective (Olympus UplanSApo 60×, 1.2 numerical apertures).

After rejecting the scattered laser light using a holographic notch filter at 532 nm (HNPF-532.0, Kaiser Optical Systems, Inc., USA), the imaging area was defined using a vertical slit located at the imaging plane of the microscope. The emission was subsequently collimated using a 20-cm focal length achromat lens (China Daheng Group, Inc.), was split by a long pass extended reflection dichroic mirror at 610 nm (610DCLP, Chroma), was recombined using an identical dichroic mirror after reflecting off a mirror each, and was finally imaged onto the ICCD using another 20-cm focal length achromat lens. To further reduce the cross-talk of signals, two band-pass filters were used in the two signal channels for Cy3 (S568/50M, Chroma) and Cy5 (D675/50M, Chroma, USA). Donor Cy3 and acceptor Cy5 images were laterally displaced and each occupied one half of the EMCCD. Each fluorescent spot in the donor channel has a corresponding one in the acceptor channel, coming from the same DNA in the sample. All images were acquired with MetaMorph software (Universal Imaging).

In the experiments, the sample cell was first fixed on the stage of the microscope. Then streptavidin (Roche) was added followed by the addition of 30–50 pM biotinylated Cy3- and Cy5-labelled DNA substrate. After washing off the unbound DNA, immobilized DNA was imaged in the unwinding buffer. To reduce the photobleaching effect, an oxygen scavenger system (0.1 mg/ml glucose oxidase, 0.02 mg/ml catalase, 1% b-mercaptoethanol and 0.4% (w/w) b-D-glucose) was used to increase the photo-stability of the fluorophores. Unwinding reaction was initiated by the simultaneous addition of RecQ and ATP.

**Mutants and their characterizations**

1. Determination of the mutation sites

PyMOL^TM^ (1.3.X) software package was used for predication of amino acids which interact with the duplex part of DNA substrate (Reference for crystal structure data: Crystal Structure of the Bloom Syndrome Helicase Blm in Complex with DNA (Newman, J.A. *et al*., 2013, DOI:[10.2210/pdb4cgz/pdb](http://dx.doi.org/10.2210/pdb4cgz/pdb))). Finally, 3 candidates (THR1110, SER1121 and LYS1125) were chosen.

1. Plasmid construction and site-directed mutagenesis

The BLM helicase core consisting of amino acid residues 642-1290 was produced by inserting the corresponding gene between the NdeI and XhoI sites of the expression plasmid pTWIN1 (New England Biolabs). The plasmid pTWIN1-BLM^642-1290^ was used as the template for site-directed mutagenesis. Specifically, amino acids THR1110, SER1121 and LYS1125 were mutated to GLY, ALA and ALA respectively. All point mutations were created by ‘splicing by overlap extension” with the mutagenic primers (Table S4). The PCR fragments were sequenced by the dideoxy method performed by AuGCT (AuGCT, China).

1. Protein expression and purification

The plasmid pTWIN1 carried different mutations were transformed into *E.coli* strain BL21 (DE3). A single colony was inoculated in 5 ml LB medium containing 100 μg/ml ampicillin and grown overnight at 37 ℃. 0.3 ml of this culture was added into 3 L LB. When the OD_600_ of culture reached about 0.6, the isopropyl-β-D-thiogalactoside was added to a final concentration of 0.3 mM and the culture was incubated at 18 ℃ for 18h to induce protein expression. The cell culture was centrifuged to harvest and resuspended in 70 ml of the column buffer (50 mM Tris-HCl pH8.0, 500 mM sodium chloride, 10% glycerol, 1 mM EDTA). Cells were breaked by passage through a French pressure cell and sonicated. The lysate was centrifuged twice at 15,000 g for 30 min at 4 ℃ and the supernatant was passed through a 0.45 μm filter before loading. The following purification procedures were performed at 18 ℃. The clarified lysate was loaded onto the chitin column (1.0 ml/minute) containing 20 ml of chitin beads (New England Biolabs) which was equilibrated with 5 column volumes of column buffer. Then, the column was washed with 20 bed volumes of column buffer to thoroughly remove the unbound proteins (2.5 ml/min). Finally, the column was washed with 2.5 column volumes of cleavage buffer (column buffer containing 50mM dithiothreitol) and stopped the flow to incubate the column at 4 ℃ for 36 hours. The target protein was eluted with column buffer (55 ml) by continuing the column flow after incubation. The pooled fractions were diluted to 150 mM NaCl by dilute buffer (20 mM Tris-HCl pH7.5, 10 mM NaCl, 10% glycerol, 1 mM EDTA). Subsequently, the dilute fraction was loaded onto the 5 ml HiTrap Heparin HP column (GE) (1.0 ml/min) to further purification. The following purification procedures were conducted with FPLC system (AKTA Purifier) at 18 ℃. The column was washed with Heparin A buffer (20 mM Tris-HCl pH7.5, 150 mM NaCl, 10% glycerol, 1 mM EDTA, 1 mM DTT) until the UV absorbance at 280 nm became stable. Bound proteins were eluted with 150 ml linear gradient of NaCl (0 to 40% B, Heparin B buffer containing 20 mM Tris-HCl pH7.5, 1 M NaCl, 10% glycerol, 1 mM EDTA, 1 mM DTT) (1.0 ml/min). Fractions of target proteins were identified by SDS-polyacrylamide gel electrophoresis and concentrated by centrifugal ultrafiltration. The concentrations of the purified proteins were determined by the Bio-Rad dye method using bovine serum albumin as the standard. The proteins were immediately frozen in single use aliquots (10 μl) by liquid nitrogen and stored at-80 ℃.

1. Equilibrium DNA binding assay

Binding of BLM to DNA substrates was performed by a fluorescence polarization assay with Infinite F200 (TECAN) at 25 ℃. DNA substrates labeled with fluorescein only were used (18-nt ssDNA and 18-bp dsDNA, Table S5). Varying amounts of proteins were added to a 150 μl aliquot of binding buffer (25 mM Tris-HCl pH7.5, 100 mM NaCl, 1 mM magnesium chloride and 3 mM DTT) containing 5 nM DNA. Each sample was equilibrated in solution for 5 min and then the fluorescence polarization was measured. The equilibrium dissociation constants were determined by fitting to the Hill equation using Kaleida Graph software package (Synergy Software).

1. Stopped-flow fluorescence measurements

The stopped-flow assays were performed with a Bio-Logic SFM-400 mixer with a 1.5-mm × 1.5-mm cell (FC-15, Bio-Logic) and the Bio-Logic MOS450/AF-CD optical system equipped with a 150-watt mercury-xenon lamp. Fluorescein was excited at 492 nm (2-nm slit width), and its emission was monitored at 525 nm using a high pass filter with 20-nm bandwidth (D525/20; Chroma Technology Co.). Unwinding kinetics were measured in a two-syringe mode, where protein and DNA substrates were pre-incubated at 37 ℃ in syringe 3 for 5 min while ATP was in syringe 4. The unwinding reaction was initiated by rapid mixing. All concentrations listed were after mixing. For converting the output data from volts to percentage unwinding, a calibration experiment was performed in a two-syringe mode, where helicase and hexachlorofluorescein-labeled single-stranded oligonucleotides were in syringe 3 while ATP with fluorescein-labeled single-stranded oligonucleotides was in syringe 4. The fluorescent signal of the mixed solution from the two syringes corresponded to 100% unwinding. The stopped-flow temperature was controlled by means of an external thermostated water bath (Ministat 125; Huber) and a high flux pump to circulate the water between the bath and the stopped-flow apparatus. The standard reaction was conducted with 4 nM DNA substrates (16 bp, 20 nt, below) and 60 nM helicases in unwinding buffer (25mM Tris-HCl pH7.5, 100 mM NaCl, 1 mM MgCl_2_, 3 mM DTT) at 37 ℃. The kinetic unwinding data curves represented averages of over 10 individual traces.

**Analysis of unwinding distributions**

Histograms of the unwinding time were fitted with the following equation from single-enzyme dynamic theory:

$f(t)=\frac{k_{1}k_{2}}{2a}\left(\exp\left[(a+b)t\right]-\exp\left[(b-a)t\right]\right)$ (1)

where

$a=\sqrt{\frac{1}{4}\left(k_{1}+k_{-1}+k_{2}\right)^{2}-k_{1}k_{2}}$, $b=-\frac{1}{2}\left(k_{1}+k_{-1}+k_{2}\right)$

and the parameters *k*_1_, *k*_-1_ and *k*_2_ describe the reaction steps in Scheme 1 in the main text.

Histograms of unwinding length of enzymes were fitted with equation 2 which is directly derived from Equation 1:

$f(l_{unwind})=\frac{k_{1}k_{2}}{2a}\left(\exp\left[(a+b)\frac{l_{unwind}}{R_{unwind}}\right]-\exp\left[(b-a)\frac{l_{unwind}}{R_{unwind}}\right]\right)$ (2)

where *R_unwind_* is the average unwinding rate (in unit of bp/s) of the helicase and *l_unwind_* /*R_unwind_* corresponds to the unwinding time *t* in Equation (1).

**Table S1** Oligonucleotide sequences for the preparation of 270-bp hairpin substrate

| **Name** | **sequence** |
| --- | --- |
| **H1** | 5'P-tgattgttgctgagg*^a^*tattgccgacagtctattccggagaggatgaatgacgtgacaggaagaacttgccagacggatggtgatgtcgagaactttatgaaaacccacgttgagccgacttatcgtgataatccgtcgtgacggacgctggccccgtggtatggcaaactcaccatgaagcgtttcactaatgggcgtggcttctggtgcctgggcggtaaagcggcctcagccatagtctcctcagcacaagtatcctcagccggatgacccctccagcgtgttttatctctgcgagcataatgcctgcgtcatccgccagtcagagctggactttactgatgcagctggctgcacgtgcggcactgcaggtgta |
| **H2** | 5'Dig-agaccacggacgcaagttcaccactcctgcgtcggcgcagaagggcttcgccatgtaccgggctattgaaatccagctccagttgcgatgatgactatgccggtatctgcaccggtggtcgtgtgctggcggcgctgacgctcgaccgtgaaatcacgcatagctcctccggtaccgcgctgaatagcctggttgacggcaatgcttggagactatggc |
| **Seal1** | 5'P-gggagcactacgttcggactagtgtactctgacttgagacttttgtctcaagtc |
| **Seal2** | 5'P-agagtacactagtccgaacgtagtgctccctgattgttgc |

*^a^* The nicking sites of Nb.BsrDI and Nb.BbvCI aer shown in color red.

**Table S2** Oligonucleotide sequences for the preparation of 40-bp and 45-bp hairpin substrates

| **Name** | **sequence** |
| --- | --- |
| **HS1** | 5'P-ggacgagtgtactctgacttgagacttttgtctcaagtc |
| **HS2** | 5'P-agagtacactcgtcc gccatagtctccaag (20t) gaacgtccaggtgta |
| **HS3** | 5'P-gacttgagacttttgtctcaagtc gccatagtctccaag cattgccgtcaaccaggctagaacgtccaggtgta |
| **HS4** | 5'Biotin-tttttacacctggacgttc |

**Table S3** Oligonucleotide sequences for the preparation of the forked DNA

| **Name** | **sequence** |
| --- | --- |
| **FK1** | ttttttttttttttttttttttttt(t*)*^a^*gtactacagctacagtgctagtacgacatgctagcagtcg-Biotin |
| **FK2** | cgactgctagcatgtcgtactagcactgtagctgtagtac(t**)*^a^*tttttttttttttt |

*^a^* t* was labelled with Cy5 and t** was labelled with Cy3.

**Table S4** Oligonucleotide sequences for site-directed mutagenesis

| **Name** | **Sequnce** |
| --- | --- |
| **hBLM642-F** | ggaattcatatggagcgtttccaaagtcttagttttcct |
| **hBLM1290-R** | gccctcgagcgatgtccattcagagtatttctgtaatac |
| **BLM T1110G-F** | CtggaagatttGGTatgaatatgctggtcg |
| **BLM T1110G-R** | cgaccagcatattcatACCaaatcttccaG |
| **BLM S1121A-F** | CattttcttggggGCTaagagtgcaaaaatcc |
| **BLM S1121A-R** | ggatttttgcactcttAGCccccaagaaaatG |
| **BLM K1125A-F** | AgtaagagtgcaGCAatccagtcaggta |
| **BLM K1125A-R** | tacctgactggatTGCtgcactcttacT |

**Table S5** Oligonucleotide sequences for DNA binding assay

| **Substrate** | **Length** | **Sequence** |
| --- | --- | --- |
| **F18** | 18nt | gcctcgctgccgtcgcca-F*^a^*-3’ |
| **F18 bis** | 18nt | 5’-tggcgacggcagcgaggc |

*^a^* F is fluorescein.

**Table S6** Oligonucleotide sequences for stopped-flow DNA unwinding assay

| **Substrate** | **Length** | **Sequence** |
| --- | --- | --- |
| **D3F16B** | 16nt | ctctgctcgacggatt-F-3’ |
| **D5H16B-20** | 36nt | 5’-HF*^a^*-aatccgtcgagcagagtttttttttttttttttttt |

*^a^* HF is hexachlorofluorescein.

**Figure S1** Mechanical properties of the DNA construct with 40-bp hairpin. The extension of the 40-bp hairpin hops frequently when the external force is adjusted to about 14 pN. The high values of the DNA extension correspond to the hairpin being fully unzipped, while the low values correspond to the hairpin being fully paired.

**Figure S2** Time trace of unwinding of the DNA with a 40-bp hairpin. The unwinding bursts consist of elementary unwinding signals. The time interval between the two bursts is about 20 seconds.

**Figure S3** The DNA construct that mimics a stalled replication fork. After tethering to the glass surface, a stretching force of about 16 pN is applied on the magnetic bead to unzip the hairpin completely. Thus the 5' flap (blue) and 3'ssDNA segment (red) anneal with each other above the lower handle. Then the stretching force is reduced to about 10 pN, the hairpin reforms while leaving a 3' single-stranded gap (blue) for the binding of BLM. This DNA structure resembles a stalled replication fork.

**Figure S4** Oscillation of DNA extension when the mimic stalled fork is unwound by BLM. (**A**) The DNA extension may continuously increase after it returns to the baseline from below, or it may decrease again. These processes may repeat for several times. (**B**) From the very beginning of the unwinding burst, the DNA extension starts to decrease below the baseline, and this may repeat for several times.


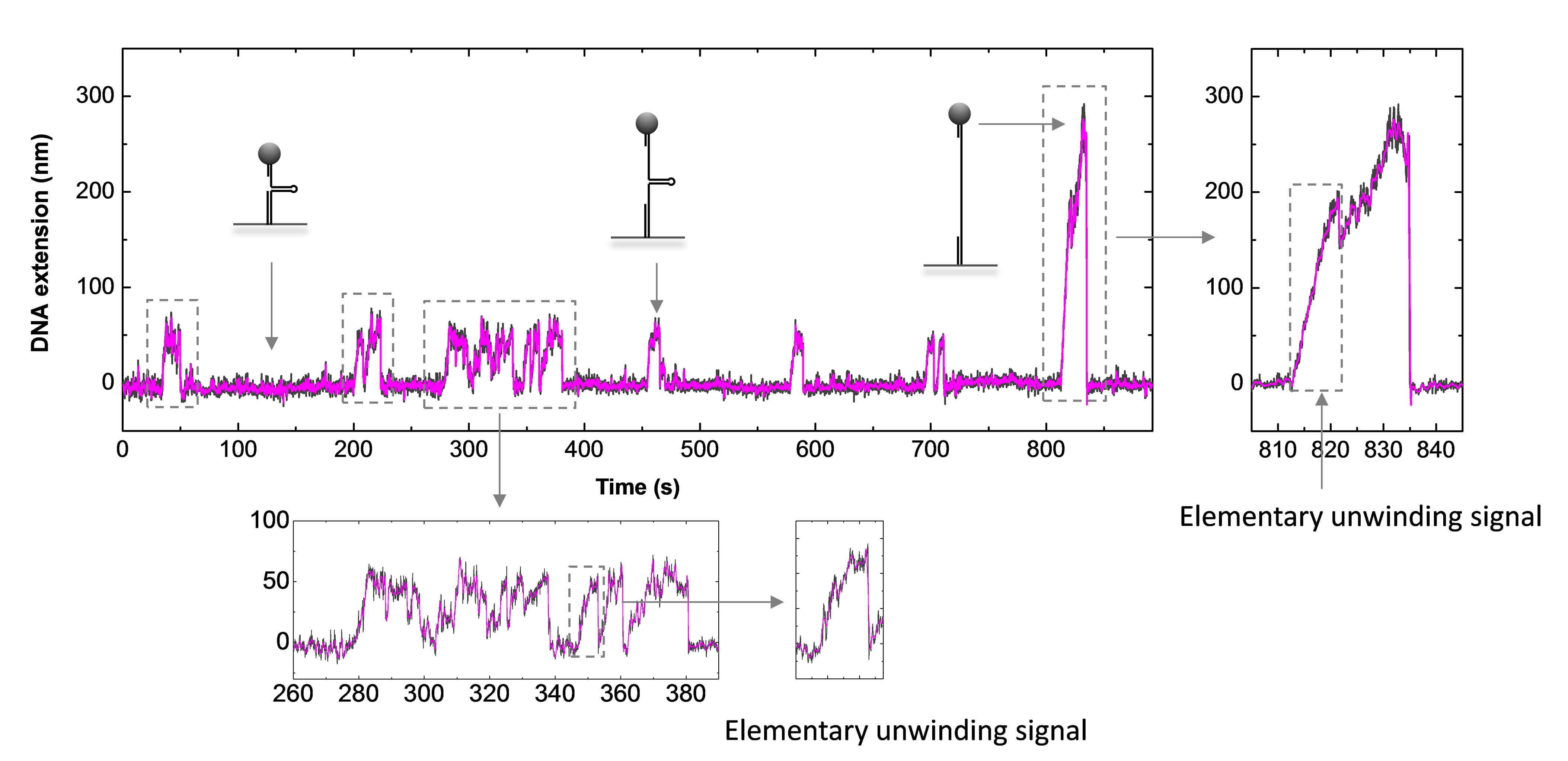


**Figure S5** Time trace of unwinding of the DNA with a 270-bp hairpin. The DNA extension is monitored in a relatively long time. It displays several bursts consisting of elementary unwinding signals. In most cases, the enzyme unwinds the substrate partially, and the elementary unwinding signal may repeat for several times in each burst (bottom panel). Occasionally, the enzyme unwinds the substrate until the 270-bp hairpin is unzipped completely in a burst that consists of elementary signals (right panel).

**Figure S6** Fast rezipping is interrupted by slow rezipping. DNA extension versus time in the presence of 5 nM BLM and 1 mM ATP. The abrupt drop of DNA extension (i.e., fast rezipping process) is interrupted by a slow rezipping event (dark gray).

**Figure S7** Distributions of on-time and off-time at two different BLM concentrations. (**A**, **B**) The BLM concentration is 5 nM (t_on_ = 25.5 ± 3.6 s, n = 197 events for A, and t_off_ = 62.9 ± 9.5 s, n = 196 events for B). (**C**, **D**) The BLM concentration is 10 nM (t_on_ = 21.5 ± 0.3 s, n = 193 events for C, and t_off_ = 20.7 ± 1.0 s, n = 194 events for D). The external force is 10 pN. The lines are best fits of the data using a single-exponential function. The corresponding decay time constants obtained from the fits are given.

**Figure S8**  Distributions of unwinding parameters for BLM-WT at an external force of 9 pN. The enzyme and ATP concentrations are 5 nM and 1 mM, respectively. (**A**) Distribution of unwinding time, which was fitted with Supplementary Equation (1), yielding *k*_1_ = 2.36 ± 0.15 s^−1^, *k*_−1_ = 0 s^−1^, *k*_2_ = 2.89 ± 0.22 s^−1^ (n = 1902 events). (**B**) Distribution of unwinding length, which was fitted with Supplementary Equation (2), yielding *k*_1_/*R_unwind_* = 0.070 ± 0.004 bp^-1^, *k*_-1_/*R_unwind_* = 0 bp^-1^ and *k*_2_/*R_unwind_* = 0.252 ± 0.069 bp^-1^ (n = 1902 events). (**C**) Distribution of unwinding rate, which was fitted with the Gaussian equation, yielding an unwinding rate of 36.5 ± 1.4 bp/s (n = 1902 events).

**Figure S9** DNA unwinding with single-molecule FRET assay. (**A**) Schematic illustration of the structure of the DNA substrate used in our experiment. The DNA substrate is tethered to the PEG-coated glass surface with biotin. (**B**) Typical time traces (100 ms exposure time) for the fluorescence emissions of Cy3 and Cy5 (upper panel) when the forked DNA was unwound by BLM, and that for the corresponding FRET (lower panel). (**C**, **D**) Histograms of ΔFRET at 5 nM BLM and 1 mM ATP (n = 329 events) (**C**) and at 5 nM BLM and 5 μM ATP (n = 276 events) (**D**).

**Figure S10** Distributions of on-time for S1121A (**A**) and K1125A (**B**). The enzyme concentration was 5 nM. Single-exponential fittings yield a time constant (t_on_) of 16.8 ± 1.1 s for S1121A (n = 269 events) and 6.2 ± 0.2 s for K1125A (n = 388 events), respectively.

**Figure S11** Distributions of unwinding parameters for S1121A at 5 nM enzyme, 1 mM ATP and 12 pN stretching force. (A) Distribution of unwinding time. It is fitted with Supplementary Equation (1), yielding ielding the rate constants of *k*_1_ = 3.03 ± 0.23s^-1^, *k*_-1_ = 0 s^-1^, and *k*_2_ = 12.84 ± 3.28 s^-1^ (n = 835 events). (B) Distribution of unwinding length. It is fitted with Supplementary Equation (2), yielding *k*_1_/*R_unwind_* = 0.074 ± 0.006 bp^-1^, *k*_-1_/*R_unwind_* = 0 bp^-1^, *k*_2_/*R_unwind_* = 0.235 ± 0.050 bp^-1^ (n = 835 events). (C) Distribution of unwinding rate. It is fitted with the Gaussian equation, yielding an unwinding rate of 45.1 ± 2.0 bp/s (n = 835 events).

**Figure S12** Distribution of the single-cycle unwinding length for K1125A at 5 nM enzyme concentration and 1 mM ATP concentration (n = 346 events).

**Figure S13** Unwinding pattern of the wild type BLM at 1 nM enzyme concentration and 1 mM ATP concentration.
